# Network approaches and applications in biology

**DOI:** 10.1371/journal.pcbi.1005771

**Published:** 2017-10-12

**Authors:** Trey Ideker, Ruth Nussinov

**Affiliations:** 1 Department of Medicine, University of California San Diego, La Jolla, California, United States of America; 2 Cancer and Inflammation Program, Leidos Biomedical Research, Inc., Frederick National Laboratory for Cancer Research, National Cancer Institute at Frederick, Frederick, Maryland, United States of America; 3 Sackler Institute of Molecular Medicine, Department of Human Genetics and Molecular Medicine, Sackler School of Medicine, Tel Aviv University, Tel Aviv, Israel

Networks have long been central to our understanding of biological systems. They are visible in the earliest delineations of cells and cell connectivity, in the immune system and the brain, in classical genetics in the form of linkage maps among genes, and between genes and phenotypes. They are the basis for our understanding of transcriptional circuits and molecular signaling and as the structure of food webs and ecosystems. More recently, the rise of social media has added a new dimension to the study of biological networks, particularly as these social networks can be explicitly charted and quantified online.

In the field of computational biology, the last 20 years have seen networks grow from a curious sideshow to a major mode of analysis. During this evolution, networks have provided both a major type of data and a conceptual framework for computation, two views that have developed in parallel with one another. The “data” view is perhaps the more straightforward, because it is clear that networks are being generated in ever increasing sizes using an expanding array of experimental techniques. As with the accrual of other experimental data in biology, the availability of large network data sets drives the creation of bioinformatics methods to analyze these data to extract biological insights.

The second view, the “network-as-concept,” has been no less powerful. Separate from the data, networks correspond to a theoretical model for representing biological structure—the graphical model—and the functional flow of information through this structure. In addition, since graphical models are core representations that arise in many different domains of engineering and physics, they greatly unify the development of computer algorithms and their application across these domains.

In this special issue, we have organized a collection of papers that address some of the most exciting opportunities and challenges for computational network biology. A potentially powerful direction is to use the knowledge of molecular networks to interpret associations between genetic variants and diseases, in which networks are not just “nice” but are likely necessary. Typically, in gene association data, an enormous number of variants can collectively influence a disease phenotype through the action of many small marginal effects, making these loci difficult to identify and even harder to biologically interpret. It is increasingly appreciated that these many genetic effects can be understood by their interrelationships within underlying transcriptional and signaling networks [1], but how to best formulate this problem is an open question. Here, the paper by Mezlini et al. [2] presents a probabilistic graphical modeling approach to this problem, and the paper by Carlin et al. [3] introduces a powerful new tool in Cytoscape for understanding the common networks impacted by a set of genetic variants through the mathematical approach of network propagation.

A second area relates to network models for the development of precision therapeutics. Many of the core issues in drug development involve network-level phenomena. Beyond their primary interaction with the target, essentially all drugs have broad effects on the cell state transmitted by metabolic, signaling, and transcriptional networks, which also integrate any off-target effects and mediate the emergence of resistance. Optimal drug target selection depends on precise understanding of the disease state and how this state can be regularized by manipulations to underlying dysregulated molecular networks. In this issue, the paper of Shen et al. [4] offers one such network-level analysis of the action of inhibitory compounds. In cancer, in particular, an attractive goal has been to develop network methodology able to understand the functional relationships among the genetic mutations driving a tumor and connecting these to secondary points of intervention to best counteract the mutational effects or exploit their weaknesses. Here, Dao and colleagues [5] present an improved analysis of genetic interactions among cancer mutations, including synergism and its opposite, mutual exclusivity.

Third, general methodology is still sorely needed to accurately detect the physical structures in the cell that are embedded in a set of molecular interaction data. Such data contain rich information that can be mined to reveal the internal organization of cellular components, whether it be the structure of proteins and protein complexes, the layout of signaling pathways, or the higher-order organization of organelles. However, there is a large gap between a list of interacting gene or protein pairs from a particular assay and a proper understanding of structure. Approaches presented by Drew et al. and Wang et al. [6, 7] serve to make this translation explicit.

Finally, this issue includes a review from Guven-Maiorov et al. [8] of the emerging need to understand the biological networks that exist among, rather than within, cells and, in particular, between hosts and their commensurate microbial communities. Construction of the molecular networks of host–microbiota can reveal cross talk and thereby help in better grasping the mechanisms of infections. Within this framework, Guven-Maiorov et al. [8] highlight structural networks, an area still in its infancy. Structural networks are powerful because they can identify microbial effectors that target distinct host proteins, providing new leads.

We hope that this special network focus will be of interest to our community, and we welcome submissions *PLOS Computational Biology* in this broad area.
